# Prevalence and Risk Factors of Discordance between Left- and Right-Hip Bone Mineral Density Using DXA

**DOI:** 10.5402/2012/617535

**Published:** 2012-06-17

**Authors:** Aziza Mounach, Asmaa Rezqi, Imad Ghozlani, Lahsen Achemlal, Ahmed Bezza, Abdellah El Maghraoui

**Affiliations:** Rheumatology Department, Military Hospital Mohammed V, P.O. Box 1018, Rabat, Morocco

## Abstract

To determine the prevalence of significant left-right differences in hip bone mineral density (BMD), and the impact of this difference on osteoporosis diagnosis, we measured bilateral proximal femora using dual energy X-ray absorptiometry (DXA) in 3481 subjects (608 males, 2873 females). The difference between left and right hip was considered significant if it exceeded the smallest detectable difference (SDD) for any of the three hip subregions. Contralateral femoral BMD was highly correlated at all measuring sites (*r* = 0.92–0.95). However, significant left-right differences in BMD were common: the difference exceeded the SDD for 54% of patients at total hip, 52.1% at femoral neck, and 57.7% at trochanter. The prevalence of left-right differences was greater in participants >65 years. For 1169 participants with normal spines, 22 (1.9%) had discordant left-right hips in which one hip was osteoporotic; for 1349 patients with osteopenic spines, 94 (7%) had osteoporosis in one hip. Participants with BMI < 20 kg/m^2^ were more likely to show major *T*-score discordance (osteoporosis in one hip and normal BMD in the other). Multiple regression analysis showed that the only significant statically parameter that persists after adjusting for all potential confounding parameters were age over 65 years.

## 1. Introduction 

Dual-energy X-ray absorptiometry (DXA) is recognized as the reference method to measure bone mineral density (BMD) accurately and reproducibly. The World Health Organization (WHO) has established DXA as the best densitometric technique for assessing BMD in postmenopausal women and based the definitions of osteopenia and osteoporosis on its results. DXA allows accurate diagnosis of osteoporosis, estimation of fracture risk, and monitoring of patients undergoing treatment [1, 2]. Hip fractures have substantially greater morbidity, mortality, and economic cost than fractures of the spine and wrist [3]. BMD of the femoral neck is a stronger predictor of hip fractures than measurement of the spine or radius and bone experts emphasize bone mineral area density (BMD in g/cm^2^) measurements of the proximal femur and spine, where trabecular bone loss is accelerated and where fractures occur [4]. Although the BMDs at different anatomic regions are correlated, the agreement between sites is low when it comes to classifying individual subjects as osteoporotic or not. Thus, *T*-score discordance between the lumbar spine and hip testing sites is a commonly observed phenomenon in densitometry [5, 6]. *T*-score discordance is the observation that the *T*-score of an individual patient varies from one key measurement site to another. Indeed, artifacts such as osteoarthritis and osteophytic calcifications of the lumbar spine influence spine BMD measurements [7]. Masud et al. [8] found that 44% of women with a mean age of approximately 70 yr had moderate or severe osteophytes. Liu et al. [9] found that in individuals older than 60 yr of age, 75% of women and 61% of men had some evidence of osteophytes.

Currently, in many central DXA studies, only one hip is scanned instead of the bilateral hips for analysis. Several studies have previously shown a high correlation between the two sides (*r* > 0.9) [10–13]. Despite this, some of these reports recommend measuring both femora. The question that arises is what effect does measurement of the contralateral hip have on osteoporosis diagnosis and treatment especially if the spine is not included in the DXA analysis? 

The aim of our study was to measure the prevalence of significant left-right differences in hip BMD, and to determine how it impacted the classification of bone status as normal, osteopenia, or osteoporosis. The difference in left-right hip BMD was considered significant if the absolute difference in left-right BMD exceeded the measure error expressed as the smallest detectable difference (SDD; the smallest number in g/cm^2^ that is statistically significantly different) for hip subregions (total hip, femoral neck, trochanter). In women with spine *T*-scores of −2.5 or below, discrepancy between hips would be of no consequence, as the classification would be “osteoporosis” regardless of hip *T*-scores. However, in women with “normal” or “osteopenic” spine values, the diagnosis of osteoporosis might be determined by hip *T*-score(s).

## 2. Methods

### 2.1. Study Design

This was a retrospective review of DXA data collected between 2004 to 2009 from one University Teaching Center. A considerable proportion of these cases were healthy postmenopausal women consulting spontaneously or referred by clinicians for densitometric evaluations. BMD was determined by a Lunar Prodigy Vision DXA system (Lunar Corp., Madison, WI, USA). The DXA scans were obtained by standard procedures supplied by the manufacturer for scanning and analysis. The study was approved by the local institutional review board. All subjects were fully ambulatory. Patients with neurological problems such as childhood polio or unilateral stroke affecting one extremity were excluded from the study. All BMD measurements were carried out by 2 experienced technicians. Daily quality control was carried out by measurement of a Lunar phantom. At the time of the study, phantom measurements showed stable results. The phantom precision expressed as the CV (%) was 0.08. Moreover, reproducibility has been assessed recently in clinical practice and showed a smallest detectable difference (SDD) of 0.040 g/cm^2^ (spine), 0.025 (trochanter), 0.024 (total hip), and 0.024 (femoral neck) [14, 15]. The measurement error is calculated using Bland and Altman's 95% limits of agreement method. In this case, where there are two observations for each subject, the standard deviation of the differences (SDdiff) estimates the within variability of the measurements. Most disagreements between measurements are expected to be between limits called “limits of agreement” defined as *d* ± *z* (1 − *a*/2) SDdiff, where *d* is the mean difference between the pairs of measurements and *z* (1 − *a*/2) is the 100 (1 − *a*/2)th centile of the normal distribution. The value *d* is an estimate of the mean systematic bias of measurement 1 to measurement 2. *d* is expected to be 0 because a true change in BMD is not assumed to occur during the interval between the two BMD measurements. Defining *a* to be 5%, the limits of agreement are +1.96 SDdiff and −1.96 SDdiff. Thus, about twice the standard deviation (SD) of the difference scores gives the 95% limits of agreement for the two measurements by the machine. A test is considered to be capable of detecting a difference, in absolute units, of at least the magnitude of the limits of agreement.

Patient BMD was measured at the lumbar spine (anteroposterior projection at L1–L4) and the femurs (femoral neck, trochanter, ward, and total hip). Data evaluated men and women who had lumbar spine and hip scans performed in the same scanning session. The hip data were acquired in automated bilateral scan mode. All DXA scans were reviewed by 2 of us (AM and GB) and patients were excluded if hip BMD was affected by documented pathology, artefacts, or technical issuessuch as positioning of the proximal femur for scanning. A total of 4162 subjects has been scanned at this period, 681 were excluded.

Using the Moroccan normative data for lumbar spine and hip [16], and the WHO diagnosis of osteoporosis (*T*-score ≤ 2.5) and osteopenia (−1 ≤ *T*-score < 2.5), *T*-scores were calculated for lumbar spine L1-4, left and right total hip, femoral neck, and trochanter. Bone status was determined by the lowest *T*-score and patients were classified as normal, osteopenic, or osteoporotic.

### 2.2. Statistical Analysis

The study was conducted on different steps.

- In the first step, and to determine the prevalence of left-right hip differences, we determined the number of pairs in which BMD exceeded the SDD; if the difference in BMD between the left-right hip exceeded the SDD then the left and right hip BMDs were considered to be statistically significantly different. Correlation between both hips was measured using the spearman coefficient.
- Thereafter, we focused on cases, in which a diagnosis of osteoporosis would be dependent on bone status of the hip, and specifically those cases in which one side was osteoporotic but not the contralateral side. Thus, scanning only the nonosteoporotic side would fail to identify and classify the patient as osteoporotic. Our premise for data analysis was that a left-right hip difference would truly impact classification only when all other skeletal sites were normal or osteopenic. That is, if spine was osteoporotic, then there would be no need to establish osteoporosis at the hip, and even if the hip was normal, the patient would still be a candidate for treatment. Therefore, the first step in data reduction was to sort patients by spine status (normal, osteopenic, osteoporotic). Next, using *T*-scores (lowest from total hip, femoral neck, or trochanter), pairs of left-right hips were identified in which one side was normal or osteopenic and the contralateral side osteoporotic. Each patient was categorized as having one (only) of the following: concordance (osteoporosis, osteopenia, or normal BMD on both hips), minor discordance 1 (osteopenic in one hip and normal in the other hip), minor discordance 2 (osteoporotic in one hip and osteopenic in the other hip), and major discordance (osteoporosis in one hip and the other hip is in the normal range).
- To study risk factors of left-right hip discordance, patients were also sorted by sex, age categories: 50–64 years, and ≥65 years; BMI categories. For comparisons between groups we used the paired *t*-test. For comparing proportions such as the percent of cases in which left-right hip pairs exceeded the SDD, we used Chi-square approximation. For all tests, differences were considered significant at *P* < 0.05. Percentages are rounded to the nearest whole number in the text.
- Finally, a multiple regression analysis was conducted to combine all potential risk factors of significant left-right difference in hip BMD.

## 3. Results 

A total of 3481 patients were identified. The mean age was 54.9 ± 1 years, range 20 to 92 years; 82.5% were women.  Table 1 summaries the characteristics of the study population. For all participants irrespective of spine status, left-right hip BMD and *T*-scores were highly correlated for all three hip subregions: *r* values for total hip, femoral neck, and trochanter were 0.95, 0.93, and 0.92, respectively. The mean BMDs (g/cm^2^) for left versus right hips were as follows: total hip 0.916 versus 0.914; femoral neck 0.859 versus 0.865; trochanter 0.735 versus 0.733. However, despite the high left-right correlations and similar left-right mean BMDs, significant left-right differences in hip BMD were common: the left-right difference in BMD exceeded the SDD for 54% of participants at total hip, 52.1% at femoral neck, and 57.7% at trochanter (Table 2).

**Figure 1:**
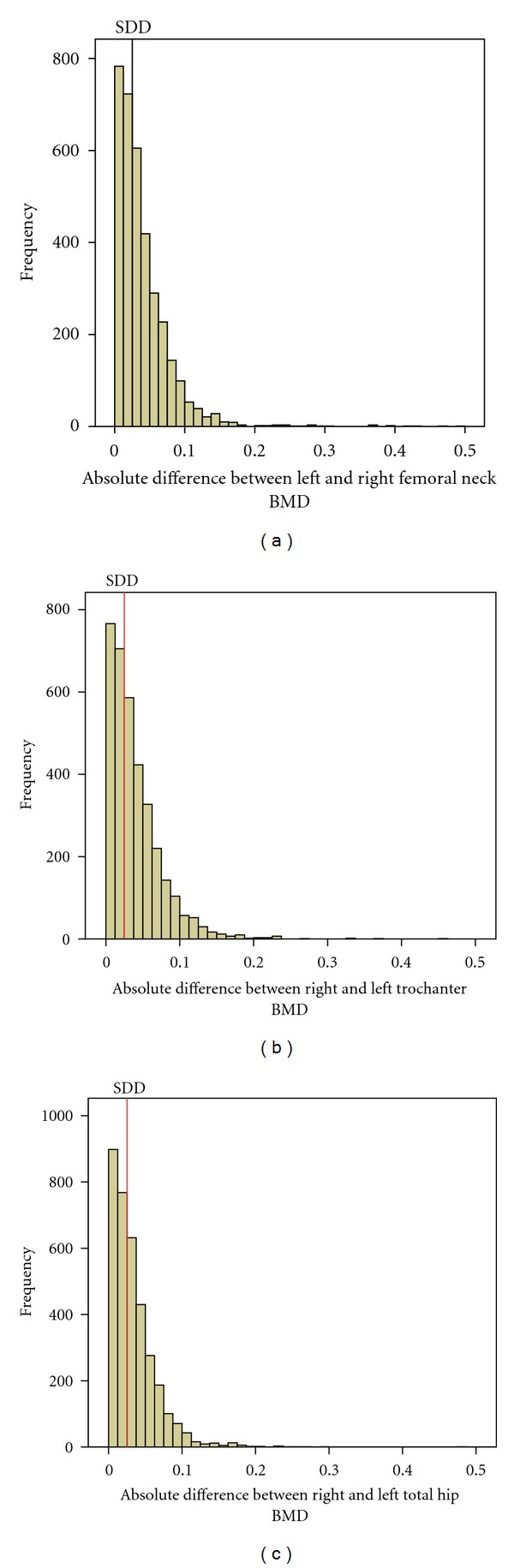
Histogram showing the frequency of absolute differences in BMD between left and right femoral neck (a), trochanter (b), and total hip (c). Numbers above the bars are the number of left-right hip pairs with the difference indicated on the abscissa. The SDD is shown as the vertical line; bars to the right show the number of hip pairs in which the left-right difference exceeded the SDD.

There was a significant effect of age, in which the prevalence of left-right differences was greater in participants >65 years compared with those participants 50–65 years (Table 3). This prevalence was not influenced by sex. Histograms show the frequency of the absolute differences between left-right BMD and the cutoff for significance (the SDD) (Figure 1). There was a high prevalence of concordance between the two hips at the three subregions (83.7% in femoral neck, 74.1% in trochanter, and 86.8% in total hip). Major discordance was seen in just two cases at the femoral neck, one case at the total hip and trochanter. For 1169 participants with normal spines, 22 (1.9%) had discordant left-right hips in which one hip was osteoporotic; for 1349 patients with osteopenic spines, 94 (7%) had osteoporosis in one hip (Table 4). For women with normal or osteopenic spines, the prevalence of major and minor discordances increased in those aged >65 years (Table 5). We studied the influence of weight on the prevalence of left-right discordance: participants with BMI < 20 kg/m² were more likely to show major *T*-score discordance (Table 6). Multiple regression analysis showed that the only significant statically parameter that persists after adjusting for all potential confounding parameters were age over 65 years (Table 7).

## 4. Discussion 

In our cohort, the prevalence of a significant left-right difference in hip BMD (defined as exceeding the SDD for any subregion) ranged from 52% to 57% depending on the compared subregion. In clinical practice, the most important is to know how much patients could be classified differently if only one hip which was normal or osteopenic was scanned but the contralateral hip was osteoporotic and not scanned. Thus, we used *T*-scores to determine the number of cases in which the left-right difference in BMD translated to left-right difference in diagnosis. Left-right hip classification discordance was more likely when the left-right hip BMD difference exceeded the SDD for any subregion. The ultimate classification difference would occur when the spine was normal or osteopenic and only one hip osteoporotic. This situation occurred in our study in 116 patients (4.6%) when the lowest *T*-score was considered. As others have suggested, the occurrence of such classifications differences is small, although statically significant. Although the percentages are low, the total number of patients affected may be large. In Hamdy et al. [17] study, for the total number of women with normal and osteopenic spines, the percent with a statistically significant left-right difference in which one hip was osteoporotic and the other osteopenic was 1% (16/1229) for normal spines and 5% (56/1159) for osteopenic spines.

Cole and Larson [18] examined the effect of dual femur densitometry on diagnosis decisions at the femur neck and total femur in 537 menopausal women (mean age 61.2 yrs). They reported left-right discordance in diagnosis classification (normal, osteopenia, and osteoporosis) with right versus left femora in 28% of subjects at one or more sites, and in 14%, 15%, and 10% of subjects at the femoral neck, trochanter, and total hip, respectively.

We looked in our study to potential risk factors of this left-right hip discordance. One final characteristic was identified in the groups of patients with osteoporosis in one hip but not the other. These patients (both sexes) were older than other patients when sorted by spine status (e.g., patients with osteoporotic hips and normal spines were more than a decade older than patients with normal spines who did not have osteoporotic hips). Increased frequency of discordance with age has been reported by several similar studies. This is a crucial observation which impacts that scanning both hips is perhaps most important for recognizing osteoporosis in elderly patients who have their first (baseline) DXA at an advanced age. Even discordance was most commonly observed in female postmenopausal patients with low BMI, these parameters (menopause, steroid use, or low BMI) were not statistically significant when a multiple regression analysis was performed. Our study is the first to describe such finding. 

Previous studies have noted a high correlation in total BMD of the contralateral hips. We also found a high correlation (*r* = 0.92–0.95) of BMD in our patients. Further, it is well-established that correlation analysis is not effective for comparing absolute values between measures. Although the correlation in BMD is quite high, small changes in BMD between contralateral hips may have a significant clinical effect when one considers the *T*-score of the lowest femur site (TH, FN, TR) for diagnosis and treatment stratification. One must keep in mind that diagnosis and treatment classification is based on the lowest *T*-score of femur sites and not the average total BMD of the hip. Several groups have noted individual variability between hips large enough to result in a different *T*-score diagnosis depending on which side was scanned [18]. The reasons for such side differences include natural genetic variation, pathology (e.g., unilateral osteoarthritis), immobilization, stroke, and so forth. It is also possible that the differences between the left and right hips are not due to true biologic differences, but rather were due to the technical skills of the operator and the reproducibility of the positioning of the patients (although this was excluded in our data) [17]. The absolute difference in BMD between hips is usually small, but can be large enough to result in diagnostic differences, especially if near thresholds between normal-osteopenia and osteopenia-osteoporosis.

Of note is that when one considers the bilateral hip mean BMD, the total area measured is greater than that with a single-hip measurement, which may improve the precision. We have already demonstrated that measuring both hips increased the reproducibility at that site [14, 15].

This study has several clinical limitations which should be noted. The first is that the subject population did not necessarily have vertebral spine artifact making the vertebral spine unsuitable for DXA analysis (this is the target population where studying left/right discordance becomes crucial as lumbar spine BMD cannot be used). The subjects were selected from a population of patients sent to an osteoporosis testing centre because they were deemed by their physician to be at risk for osteoporosis. A second limitation is the consideration that the WHO definition of osteoporosis determined at Geneva in 1994 only took the single-hip measurement into account. Subsequently, diagnosis and treatment recommendations have typically been based on a single femur measurement. Use of the bilateral hip study would alter the observed prevalence of osteoporosis, thereby jeopardizing the basic rationale for the WHO's definition of osteoporosis on the basis of BMD in postmenopausal women. Another limitation is reliance on precision assessment results from a single DXA system. Thus, the prevalence of left-right differences will be center dependent, which, although obvious, may impact the generalizability of our findings. However, this study included a high number of patients and was the first to include men and premenopausal women.

In summary, our results suggest that it is possible that a therapeutic opportunity will be missed if only one hip is scanned and it happens to be the one that is normal or osteopenic, whereas the other hip (which is not scanned) happens to be osteoporotic especially if the spine is normal or ininterpretable (e.g., osteoarthritis). This seems particularly true in elderly patients aged over 65. Moreover, the consequences of this discordance on the recently described 10-year absolute risk of fractures (FRAX) may be very important [19]. Because of ease and speed of bilateral hip measurements with modern DXA with the consequent low radiation dose, there are no longer technical barriers to scanning both hips.

## Figures and Tables

**Table 1:** Characteristics of the study population.

	Femoral neck	Trochanter	Total hip
Age (years ± SD)	54.9 ± 12.7	54.9 ± 12.7	54.9 ± 12.7
BMI (Kg/m²)	27.9 ± 45.1	27.9 ± 45.1	27.9 ± 45.1
Mean right BMD (g/cm²)	0.865	0.733	0.914
Mean left BMD (g/cm²)	0.859	0.735	0.916
Mean BMD difference (g/cm²)	−0.005	−0.021	−0.001
Mean right *T*-score	−1.169	−0.766	−0.859
Mean left *T*-score	−1.214	−0.747	0.916
Mean *T*-score difference	0.045	−0.019	−0.007

**Table 2:** Prevalence of difference between left-right hip subregions.

	Difference < SDD	Difference > SDD
Femoral neck	47.9%	52.1%
Trochanter	42.3%	57.7%

Total hip	46%	54%

**Table 3:** Percent of cases in which the left-right hip subregion exceeded the SDD with patients sorted by age.

		Femoral neck (%)	Trochanter (%)	Total hip (%)
Age	50–65 years (*n* = 1647)	815 (49.5)	945 (57.4)	844 (51.2)
	>65 years (*n* = 712)	401 (56.3^∗^)	440 (61.8^∗^)	415 (58.3^∗^)

^∗^Significantly greater than for 50–65-year group, Chi-square, *P* < 0.01 or less.

**Table 4:** Patients with osteoporotic hips sorted by spine status.

Patients with osteoporosis in hips, number (% of total, rounded)			
Spine status	Both	One	None
Normal (*n* = 1169)	17 (1.5)	22 (1.9)	1130 (96.7)
Osteopenia (*n* = 1349)	93 (6.9)	94 (7)	1162 (86.1)

**Table 5:** Discordant hip pairs in patients with normal and osteopenic spines, sorted by age and sex.

	Concordance	Minor discordance 1	Minor discordance 2	Major discordance
Males	248 (49.4)	221 (44)	22 (4.4)	11 (2.2)
Females < 50 yrs	430 (58.3)	270 (36.6)	17 (2.3)	21 (2.8)
Females 50–64 yrs	380 (38.5)	528 (53.4)	38 (3.8)	42 (4.3)
Females ≥ 65 yrs	42 (14.4)	173 (59.5)	29 (10)	47 (16.2)

Concordance: osteoporosis, osteopenia, or normal BMD on both hips, minor discordance 1: osteopenia in one hip and normal BMD in the other hip, minor discordance 2: osteoporosis in one hip and osteopenia in the other hip, and major discordance: osteoporosis in one hip and normal BMD in the other.

**Table 6:** Prevalence of diagnosis discordances between left and right hips sorted by sex and BMI.

		Major discordance	Minor discordance 2
Sample study	BMI < 20 (*n* = 176)	50 (28.4%)	13 (7.4%)
	20 < BMI < 30 (*n* = 2165)	318 (14.7%)	147 (6.8%)
	BMI ≥ 30 (*n* = 1140)	111 (9.7%)	67 (5.9%)

Females	BMI < 20 (*n* = 99)	38 (38.4%)	5 (5.1%)
	20 < BMI < 30 (*n* = 1692)	287 (17%)	114 (6.7%)
	BMI ≥ 30 (*n* = 1082)	110 (10.2%)	65 (6%)

Males	BMI < 20 (*n* = 77)	12 (15.6%)	8 (10.4%)
	20 < BMI < 30 (*n* = 473)	31 (6.6%)	33 (7%)
	BMI > 30 (*n* = 58)	1 (1.7%)	2 (3.4%)

Minor discordance 2: osteoporosis in one hip and osteopenia in the other hip; major discordance: osteoporosis in one hip and normal BMD in the other.

**Table 7:** Multiple-regression analysis of potential risk factors for significant let-right difference in hip bone mineral density.

	Difference >SDD
Age > 65 yrs	1.35 [1.07–1.71]^∗^
Menopause	0.88 [0.71–1.08]
BMI < 20	0.87 [0.53–1.43]
Corticoid use (>3 months)	0.79 [0.53–1.17]

Total hip BMD	0.25 [0.05–1.11]
